# Hesperidin and capsaicin, but not the combination, prevent hepatic steatosis and other metabolic syndrome-related alterations in western diet-fed rats

**DOI:** 10.1038/s41598-018-32875-4

**Published:** 2018-10-10

**Authors:** Andrea Mosqueda-Solís, Juana Sánchez, Bárbara Reynés, Mariona Palou, María P. Portillo, Andreu Palou, Catalina Picó

**Affiliations:** 1 Laboratory of Molecular Biology, Nutrition and Biotechnology (Nutrigenomics and Obesity group), University of the Balearic Islands, 07122 Palma, Spain; 2 Instituto de Investigación Sanitaria Illes Balears, 07010 Palma, Spain; 3 Nutrition and Obesity Group, Department of Nutrition and Food Science, Faculty of Pharmacy and Lucio Lascaray Research Center, University of the Basque Country (UPV/EHU), Vitoria, Spain; 4 CIBER Fisiopatología de la Obesidad y Nutrición (CIBEROBN), Instituto de Salud Carlos III (ISCIII), Madrid, Spain

## Abstract

We aimed to assess the potential effects of hesperidin and capsaicin, independently and in combination, to prevent the development of obesity and its related metabolic alterations in rats fed an obesogenic diet. Three-month-old male Wistar rats were divided into 5 groups: Control (animals fed a standard diet), WD (animals fed a high fat/sucrose (western) diet), HESP (animals fed a western diet + hesperidin (100 mg/kg/day)), CAP (animals fed a western diet + capsaicin (4 mg/kg/day)), and HESP + CAP (animals fed a western diet + hesperidin (100 mg/kg/day) + capsaicin (4 mg/kg/day)). Hesperidin and capsaicin were administered by gavage. Capsaicin decreased body fat gain and prevented insulin resistance, whereas hesperidin showed little effect on body fat gain and no apparent effects on insulin resistance. No additive effects were observed with the combination. Capsaicin and hesperidin, separately, improved blood lipid profile, diminished hepatic lipid accumulation, and prevented non-alcoholic steatohepatitis in western diet-fed rats, but the combination showed lower effects. Hesperidin alone, and to a lesser extent capsaicin or the combination, displayed hypotensive effects in western diet-fed rats. In conclusion, capsaicin and hesperidin, separately, exhibit health beneficial effects on metabolic syndrome-related alterations in western diet-fed rats, but the effects are mitigated with the combination.

## Introduction

Obesity has reached epidemic proportions globally. In 2016, more than 1.9 billion adults worldwide were overweight, and of these over 600 million were clinically obese^1^. Obesity is the consequence of a prolonged disruption in energy homeostasis, in which the energy gain exceeds the energy expenditure. This condition is considered as a multifactorial disease that is influenced by lifestyle, cultural, environmental, genetic, physiological and metabolic factors. Notably, the intake of western diets, characterized especially in high consume of simple carbohydrates and saturated fats, are leading to an increase in the prevalence of obesity and its related alterations, such as insulin resistance, hyperlipemia and non-alcoholic fatty liver, among others^2^. Attention of the scientific community is focused on the implementation of innovative and effective strategies for the prevention and treatment for this pathology and its comorbidities^3^.

Nowadays the use of natural bioactive compounds is trending as alternative methods for the treatment and management of obesity and related diseases, but the efficacy of such approaches depends on the absorption, metabolism and bioavailability of such compounds, which may be influenced by disease state^4^. In addition, possible interactions between bioactive agents, leading to additive or synergistic effects or, on the contrary, to a decrease in their efficacy, should be considered. These aspects may have important implications for functional food development and assessment. However, no much information is available regarding this issue. The study is of interest since the level of a single natural compound may be too low to exert sufficient beneficial effects. By contrast, the combination of compounds acting via an additive and/or synergistic mode to either the same or diverse targets may be of interest in preventing a pathological process.

For hesperidin (C_28_H_34_O_15_), a flavanone present in citrus fruit, diverse biological activities of therapeutic interest have been described, including the capacity to lower serum and liver triacylglycerols (TG) as well as anti-adipogenic, anti-inflammatory, antioxidant and insulin-sensitizing properties^5–8^. Therefore, hesperidin may be of interest to improve obesity-related disorders. In fact, several studies, including preclinical and clinical trials, have demonstrated that hesperidin may have therapeutic effects on a great variety of diseases, such as cardiovascular diseases, diabetes, cancer, and neurological and psychiatric disorders, among others^9^.

Other compounds of potential interest are the capsaicinoids (also known as capsinoids), a group of molecules naturally present in chilli peppers. The most abundant and studied is the capsaicin (trans-8-methyl-N-vanillyl-6-nonenamide (C_18_H_27_NO_3_), which is responsible for the pungent sensation^10,11^. Capsaicin is recognized for its potential anti-inflammatory, antioxidant, antimicrobial, anticancer, and antiobesity properties among others^12^. Several studies have demonstrated that capsaicin decreases body weight gain, hepatic lipid accumulation and insulin resistance induced by high-fat diet feeding^13,14^. The anti-obesity effects of capsaicin have been related in part to its capacity to stimulate the sympathetic nervous system and thus to reduce energy intake and increase energy expenditure and fat oxidation, through the effects of catecholamines^12^. However, it is not clear whether the long-term effects of capsaicin on obesity may be explained by this mechanism. It is accepted that much of the effects of capsaicin on metabolic health, particularly linked to its fat-lowering action, are caused by stimulation of the transient receptor potential cation channel subfamily V member 1 (TRPV1)^15,16^. TRPV1, also known as capsaicin receptor, belongs to the family of non-selective cation channels with high calcium permeability^17^. This is highly expressed in sensory neurons and in vasculature, adipose, and liver tissues^18,19^. TRPV1 activation has been described to result in recruitment of catecholaminergic neurons in the rostral ventrolateral medulla of the brain^20^. Capsaicin-induced calcium influx through TRPV1 channels has been shown to prevent adipogenesis and obesity in wild-type mice under high-fat diet feeding but not in TRPV1 knockout mice, indicating that TRPV1 is directly involved in these effects *in vivo*^16^.

Considering that hesperidin and capsaicin can affect lipid metabolism and induce triacylglycerol (TG)-lowering effects by different mechanisms, the combined effects of both compounds on obesity and related metabolic alterations is of interest. In this context, the aim of this study was to screen the potential effects of dietary hesperidin and capsaicin, separately, and the combination of both compounds (hesperidin + capsaicin) to prevent the development of obesity and its related metabolic alterations, particularly insulin resistance, dyslipidemia, fatty liver disease and hypertension, induced in rats by feeding a western diet.

## Results

### Body weight, food intake and circulating parameters

Results on body weight and body fat, liver weight, and food intake of the 5 groups of animals are summarized in Table 1.

**Table 1:** Weight-related parameters and food intake.

	Control	WD	HESP	CAP	HESP + CAP
Initial body weight (g)	362 ± 16	353 ± 8	351 ± 15	356 ± 11	358 ± 15
Final body weight (g)	428 ± 19	443 ± 14	433 ± 14	423 ± 15	433 ± 19
Body weight gain (3–5 months) (g)	66 ± 7^a^	91 ± 9^b^	83 ± 4^a,b^	67 ± 4^a^	75 ± 8^a,b^
Initial body fat (%)	10.8 ± 0.7	10.8 ± 0.8	10.7 ± 0.7	10.9 ± 0.4	10.9 ± 0.8
Final body fat (%)	11.5 ± 0.5^a^	18.4 ± 1.5^b^	15.9 ± 0.5^b,c^	15.1 ± 0.4^c^	15.9 ± 1.0^b,c^
Liver (g)	12.5 ± 0.7^a^	14.6 ± 0.3^b^	13.9 ± 0.5^a,b^	13.0 ± 0.4^a,c^	14.1 ± 0.5^b,c^
Cumulative food intake (3–5 months) (Kcal)	3970 ± 151^a^	4683 ± 356^b^	4431 ± 104^a,b^	4337 ± 191^a,b^	4351 ± 242^a,b^

Initial and final body weight and body fat (before and at the end of treatment, respectively) are indicated. Data are mean ± SEM. One-way ANOVA was used to determine differences between groups followed by a least significance difference (LSD) *post hoc* analysis (*P* < 0.05), a ≠ b ≠ c.

At the age of 5 months, after 8 weeks of treatment, no significant differences in body weight were found among the 5 experimental groups. However, the western diet (WD) group, but not the capsaicin (CAP) group, showed higher body weight gain than controls. Animals of the hesperidin (HESP) and HESP + CAP groups showed intermediate values (*P* < 0.05, LSD *post-hoc* analysis). Capsaicin-treated animals also displayed, at the end of the intervention period, lower body fat percentage than animals of the WD group, but higher than the control group. Body fat content in HESP and the HESP + CAP groups was slightly higher than that of the CAP group, and not significantly different from the WD group (*P* < 0.05, LSD *post-hoc* analysis). Animals in the WD group, but not animals in the CAP group, also showed higher liver weight than the controls, whereas animals treated with hesperidin or with the combination of both compounds showed intermediate values (*P* < 0.05, LSD *post-hoc* analysis). Regarding food intake, animals of the WD group showed greater cumulative energy intake than the controls, whereas WD-fed animals treated with hesperidin, capsaicin or the combination of both compounds showed intermediate values (*P* < 0.05, LSD *post-hoc* analysis).

Circulating levels of glucose, insulin, leptin, TG and non-esterified fatty acids (NEFA) under *ad libitum* and fasting conditions are showed in Table 2. Non-significant differences were found among groups in glucose and insulin levels in both conditions. However, the HOMA-IR was different among groups. Capsaicin-treated animals showed lower values with respect to the animals from the WD and HESP groups (*P* < 0.05, LSD *post-hoc* analysis). Animals of the control and HESP + CAP groups showed intermediate levels. Glucose and insulin levels were lower under fasting conditions with respect to levels in the fed state in all groups except in the HESP + CAP group (for both glucose and insulin) and in the HESP group (for insulin) (*P* < 0.05, Paired *t* test). Animals on the WD group showed greater plasma leptin levels (both under fed and fasting conditions) than control animals (*P* < 0.05, LSD *post-hoc* analysis). Notably, animals of the CAP and HESP + CAP groups showed lower fed-state leptin levels than animals on the WD group and intermediate levels between control and WD animals under fasting conditions. All groups showed lower leptin levels under fasting conditions compared to levels under feeding conditions (*P* < 0.05, Paired *t* test). Animals on the WD group showed higher circulating TG levels under feeding conditions than the control animals (*P* < 0.05, LSD *post-hoc* analysis). TG levels were partially normalised to control levels in the HESP and CAP groups, but not in the HESP + CAP group (*P* < 0.05, LSD post-hoc analysis). No significant differences were found among groups regarding TG levels under fasting conditions. However, fasting TG levels were significantly lower than fed-state TG levels in the WD and HESP + CAP groups.

**Table 2:** Blood parameters of control (C), western diet (WD), hesperidin (HESP), capsaicin (CAP), and hesperidin and capsaicin (HESP + CAP) rats at the end of treatment.

	Control	WD	HESP	CAP	HESP + CAP
Glucose (mg/dL)					
*Ad libitum*	127 ± 4	123 ± 3	119 ± 4	119 ± 2	120 ± 5
14 h fasting	100 ± 5*	104 ± 8*	100 ± 3*	97 ± 4*	107 ± 5
Insulin (μg/L)					
*Ad libitum*	1.29 ± 0.25	1.60 ± 0.24	1.56 ± 0.10	1.22 ± 0.09	1.71 ± 0.36
14 h fasting	0.83 ± 0.24*	1.07 ± 0.18*	1.10 ± 0.20	0.72 ± 0.14*	0.75 ± 0.11
HOMA-IR					
14 h fasting	3.69 ± 0.80^a,b^	7.07 ± 1.38^a^	6.77 ± 1.31^a^	3.60 ± 0.68^b^	4.93 ± 0.71^a,b^
Leptin (μg/L)					
*Ad libitum*	5.30 ± 0.45^a^	12.19 ± 1.65^b^	9.71 ± 1.04^b,c^	8.08 ± 0.59^c^	8.20 ± 0.99^c^
14 h fasting	2.08 ± 0.28^a,^*	5.11 ± 0.97^b,^*	4.35 ± 0.82^b,^*	3.37 ± 0.51^a,b,^*	3.50 ± 0.72^a,b,^*
TG (mg/mL)					
*Ad libitum*	0.730 ± 0.127^a^	1.82 ± 0.25^b^	1.39 ± 0.22^a,b^	1.39 ± 0.18^a,b^	1.8 ± 0.32^b^
14 h fasting	0.769 ± 0.124	0.717 ± 0.049*	0.905 ± 0.141	0.816 ± 0.171	0.928 ± 0.196*
NEFA (mmol/L)					
*Ad libitum*	0.498 ± 0.036^a^	0.984 ± 0.084^b^	0.781 ± 0.046^c^	0.740 ± 0.046^c^	0.765 ± 0.063^c^
14 h fasting	1.19 ± 0.11*	0.902 ± 0.058	1.11 ± 0.14*	0.978 ± 0.089	0.938 ± 0.194
AST (U/L)					
*Ad libitum*	53.0 ± 5.0	54.8 ± 3.1	54.1 ± 4.6	52.3 ± 8.3	44.2 ± 3.0
ALT (U/L)					
*Ad libitum*	9.14 ± 1.49	8.25 ± 2.33	11.8 ± 1.6	8.25 ± 1.73	8.00 ± 2.63

Data are mean ± SEM. One-way ANOVA was used to determine differences between groups under ad libitum and fasting condition separately, followed by a least significance difference (LSD) *post-hoc* analysis (*P* < 0.05), a ≠ b ≠ c. *P < 0.05 fasting *versus ad libitum* condition (Paired *t*-test). HOMA-IR: homeostatic model assessment for insulin resistance; TG: triacylglycerol; NEFA: non-esterified fatty acids; AST: aspartate transaminase (AST); ALT: alanine transaminase.

Regarding NEFA, animals on the WD group showed higher circulating levels under feeding conditions than the control animals (*P* < 0.05, LSD *post-hoc* analysis). HESP, CAP and HESP + CAP groups showed lower levels than animals on the WD group, but higher than controls (*P* < 0.05, LSD *post-hoc* analysis). No significant differences were found among groups regarding NEFA levels under fasting conditions. NEFA levels increased under fasting conditions in the control and HESP groups (*P* < 0.05, paired *t* test). No significant differences between groups were found regarding the markers of liver damage aspartate transaminase (AST) and alanine transaminase (ALT).

### Energy expenditure, locomotive activity and respiratory exchange ratio

**Figure 1:**
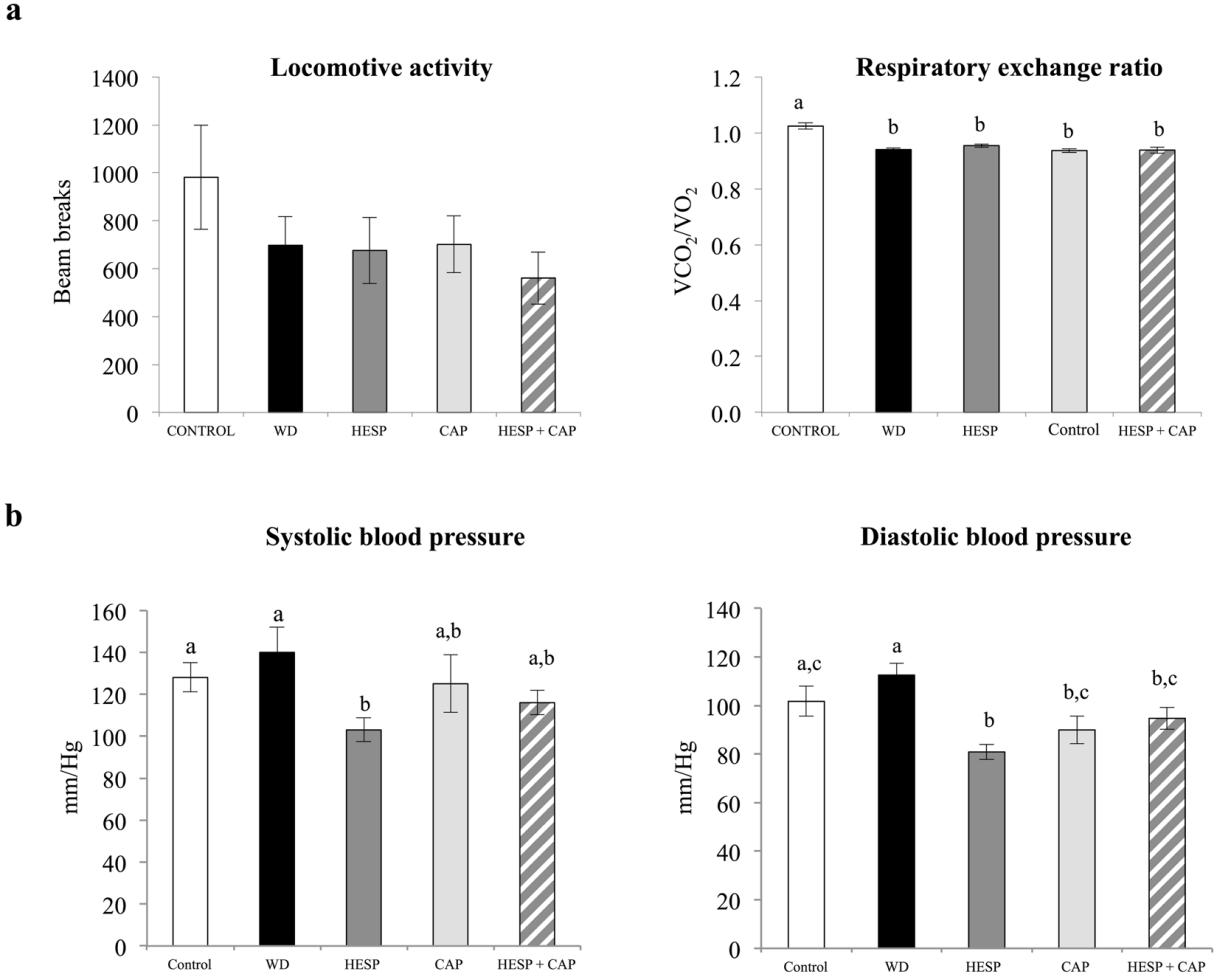
(**a**) Locomotive activity and respiratory exchange ratio (RER) after 6 weeks of treatment, and (**b**) systolic and diastolic blood pressure after 5 weeks of treatment, of control (C), western diet (WD), hesperidin (HESP), capsaicin (CAP), and hesperidin and capsaicin (HESP + CAP) groups. Data are mean ± SEM (n = 7–8). Statistical analysis between groups was performed by one-way ANOVA, followed by LSD post-hoc analysis (*P* < 0.05), a ≠ b ≠ c.

No differences were found concerning energy expenditure at the age of 5 months (data not shown). Animals fed a western diet showed a trend to lower locomotive activity compared to controls, but differences were not significant (Fig. 1a). Respiratory exchange ratio (RER) was decreased in all western diet-fed groups with respect to controls (*P* < 0.05, LSD *post-hoc* analysis), but no differences were found among them (Fig. 1a).

### Blood pressure

Animals fed a western diet and treated with hesperidin showed lower systolic and diastolic blood pressure than western diet-fed animals and even the control animals (*P* < 0.05, LSD *post-hoc* analysis) (Fig. 1b). Values of diastolic blood pressure in the control group were somewhat higher than those usually described in Wistar rats^21^, but within the same range as those described by other authors^22^. The treatment with capsaicin or the combination of hesperidin and capsaicin was also associated with decreased values of diastolic blood pressure, and a trend to lower systolic blood pressure, reaching values not significantly different from controls.

### Hepatic lipid content and histological analysis

**Figure 2:**
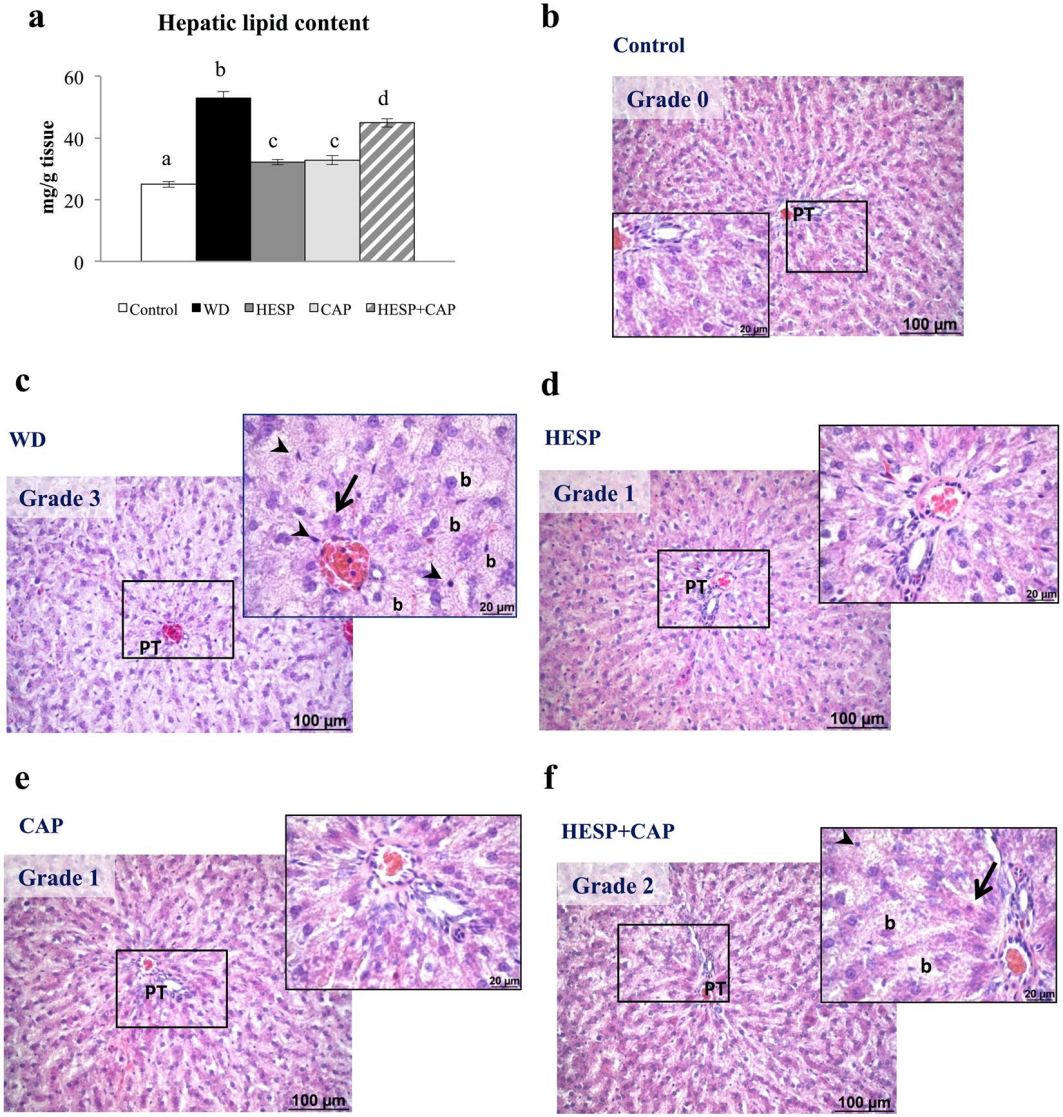
(**a**) Hepatic lipid content, and (**b**–**f**) representative liver slides stained with hematoxylin/eosin (x20) and numerical grading of hepatic steatosis in liver of control (C), western diet (WD), hesperidin (HESP), capsaicin (CAP), and hesperidin and capsaicin (HESP + CAP) groups at the end of treatment. In a), data are mean ± SEM (n = 7–8). Statistical analysis between groups was performed by one-way ANOVA, followed by LSD post-hoc analysis (*P* < 0.05), a ≠ b ≠ c ≠ d. Symbol in images: PT: portal triad, b: ballooned hepatocytes, arrow: necrotic hepatocyte, arrowhead: lymphocytes.

Figure 2a shows the hepatic lipid content of animals after the intervention period. Animals of the WD group displayed a significant increase (112%) in the lipid content with respect to controls. Notably, western diet-fed animals treated with either hesperidin or capsaicin separately and, to a lesser extent with the combination, showed significantly lower hepatic lipid content with respect to the WD group (39%, 38%, and 15% decrease, respectively). Nevertheless, all western diet-fed groups displayed higher hepatic lipid content than controls (*P* < 0.05, LSD *post-hoc* analysis). Liver histological analysis (Fig. 2b–f) revealed no signs of steatosis in the control animals. However, western diet-fed groups presented different degrees of hepatic steatosis: grade 1 (HESP and CAP groups), grade 2 (HESP + CAP group), and grade 3 (WD group). Moreover, the WD and HESP + CAP groups exhibited the presence of hepatocyte ballooning, necrotic hepatocytes and infiltrated lymphocytes, which are indicative characteristics of non-alcoholic steatohepatitis (NASH), and distinguished from simple steatosis^23^. These results suggest that hesperidin and capsaicin separately, but not the combination, can prevent the development of NASH induced by western diet feeding.

### mRNA and protein expression of energy metabolism-related genes in liver

**Figure 3:**
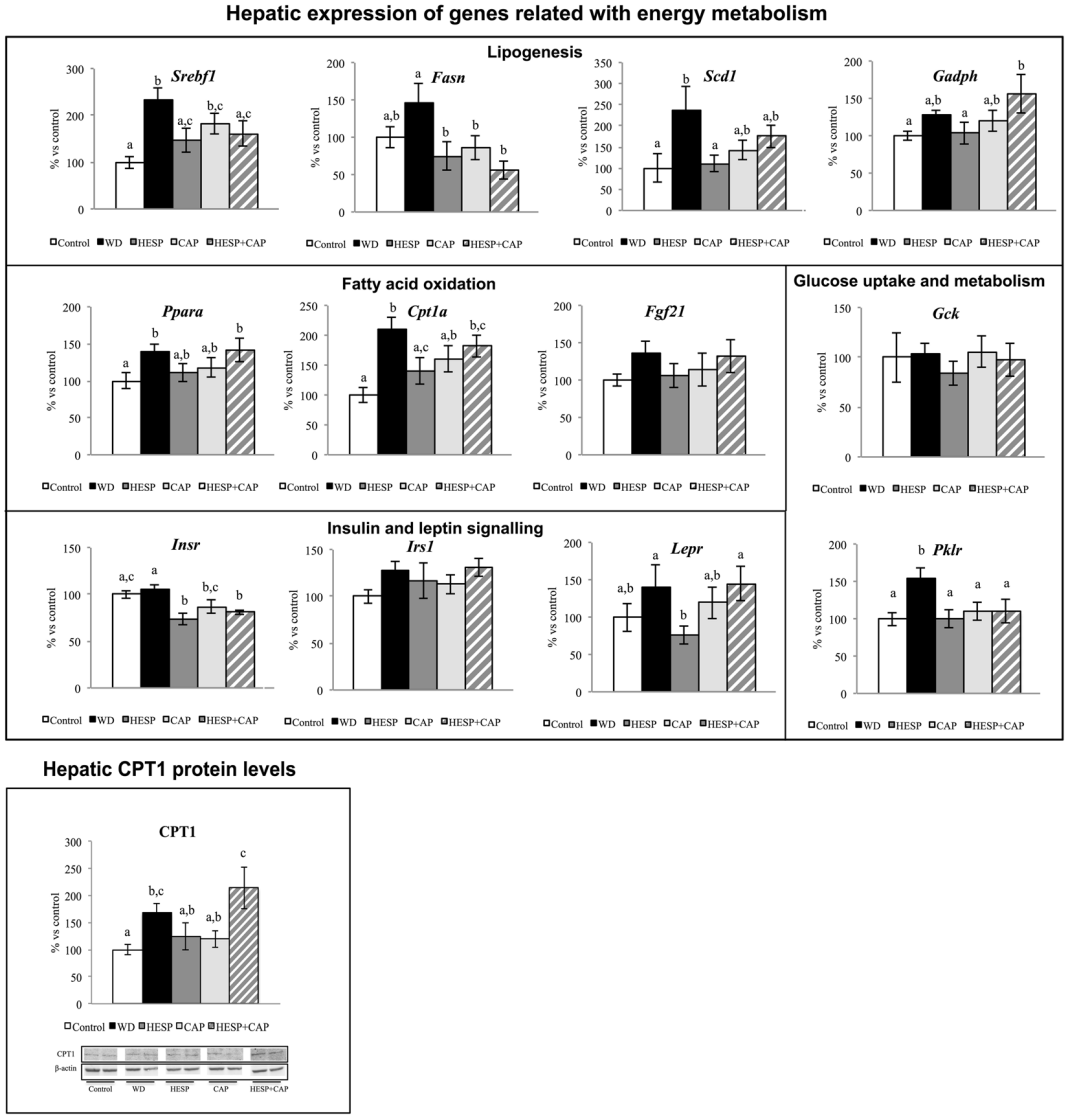
Expression of genes related to energy metabolism (lipogenesis, fatty acid oxidation, glucose uptake and metabolism, and insulin and leptin signaling) and CPT1 protein levels in liver of control (C), western diet (WD), hesperidin (HESP), capsaicin (CAP), and hesperidin and capsaicin (HESP + CAP) rats at the end of treatment. mRNA levels were measured by real-time PCR. Specific CPT1 levels were measured by western blot. Representative bands for CPT1 and β-actin (deriving from the same gel) are shown. mRNA and protein levels were expressed as a percentage of the control group. Data are mean ± SEM (n = 7–8). Statistical analysis between groups was performed by one-way ANOVA, followed by LSD post-hoc analysis (*P* < 0.05), a ≠ b ≠ c.

mRNA and protein expression levels of selected genes related to energy metabolism in liver in the different groups of animals are summarized in Fig. 3.

WD group presented higher expression levels of the lipogenesis-related genes *Srebf1*, and *Scd1* with respect to controls (*P* < 0.05, LSD *post-hoc* analysis). The increase was prevented in hesperidin-treated animals and, in the case of *Srebf1*, in animals treated with the combination of both molecules. Animals treated with capsaicin or the combination showed a trend to lower *Scd1* mRNA expression levels than western diet-fed animals, but not different from the controls (*P* < 0.05, LSD *post-hoc* analysis).

Animals treated with hesperidin, capsaicin or the combination also showed lower expression levels of *Fasn* with respect to animals of the WD group (*P* < 0.05, LSD *post-hoc* analysis), whereas the control animals showed intermediate levels. Animals treated with the combination displayed greater expression levels of *Gadph* than the controls and heperidin-treated animals, whereas the WD and CAP groups exhibited intermediate levels (*P* < 0.05, LSD *post-hoc* analysis).

Regarding fatty acid oxidation-related genes, the WD group showed increased expression levels of *Ppara* and *Cpt1a*, whereas animals of the HESP and CAP groups showed decreased (or a trend to decreased) expression levels, reaching values not different from controls. However, this trend was not observed in animals treated with the combination, which exhibited expression levels similar to the WD group. A similar pattern was observed for mRNA expression levels of *Fgf21*, but differences among groups were not significant. Changes between groups regarding specific CPT1 protein levels were similar to those observed for transcript levels. Animals treated with the combination displayed higher CPT1 levels than animals of the control, HESP and CAP groups (*P* < 0.05, LSD *post-hoc* analysis).

The expression level of the glucose metabolism related gene *Pklr*, was significantly increased in the WD group with respect to controls (*P* < 0.05, LSD *post-hoc* analysis). Notably, hesperidin and capsaicin, individually or the combination of both, normalised their expression to the control levels. No significant differences were found regarding *Gck* expression levels among groups.

Regarding insulin and leptin signalling, the HESP, CAP and HESP + CAP groups showed lower expression levels of *Insr* than control and WD groups, with no significant differences among them (*P* < 0.05, LSD *post-hoc* analysis); however, no significant changes were observed in *Irs1* expression among groups. In turn, the HESP group showed decreased *Lepr* mRNA expression levels than the WD and the HESP + CAP groups, while the controls and animals treated with capsaicin showed intermediate levels (*P* < 0.05, LSD *post-hoc* analysis).

**Figure 4:**
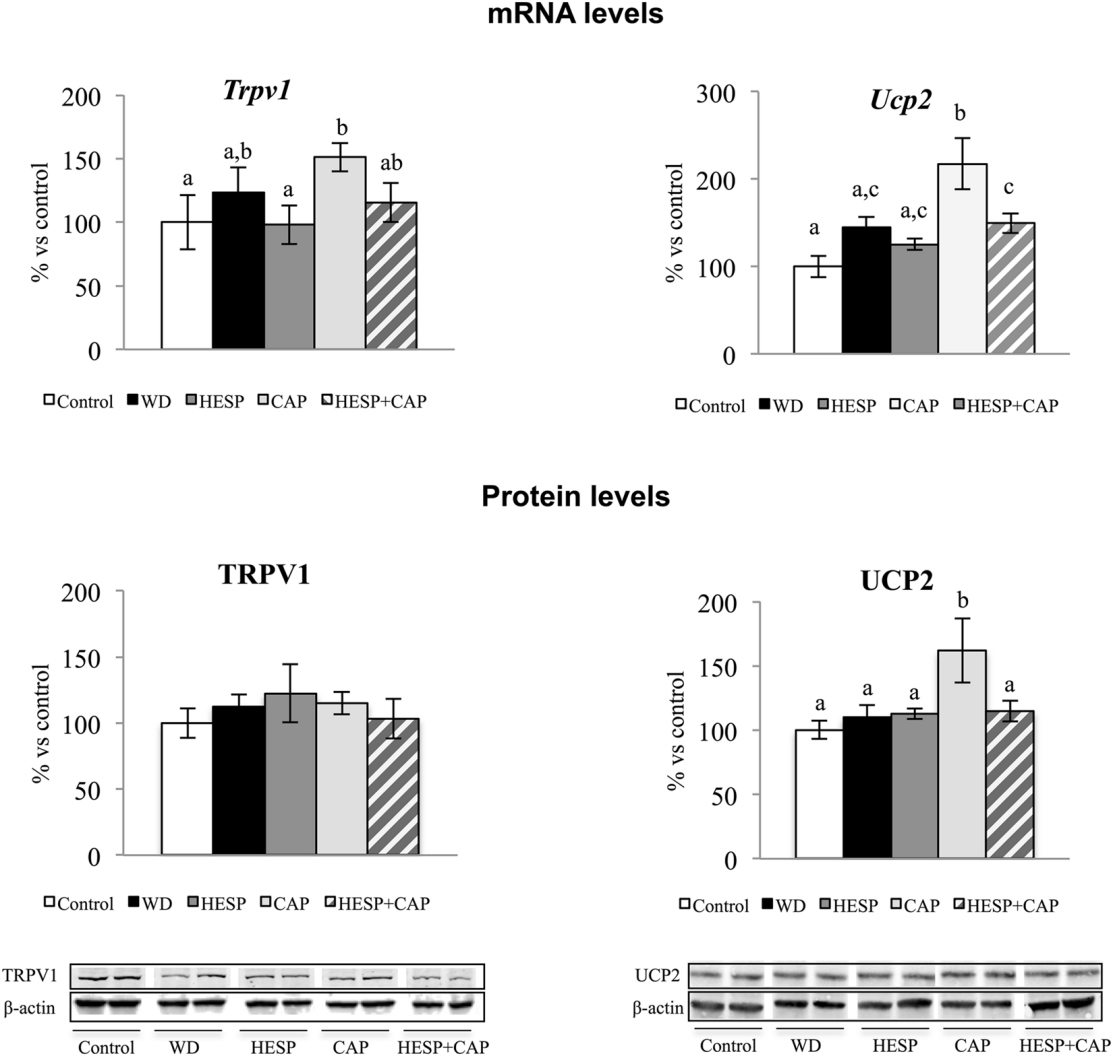
mRNA expression levels and protein levels of *Trpv1* and *Ucp2* in liver of control (C), western diet (WD), hesperidin (HESP), capsaicin (CAP), and hesperidin and capsaicin (HESP + CAP) rats at the end of treatment. mRNA levels were measured by real-time PCR. Specific protein levels were measured by western blot. Representative bands for TRPV1 and UCP2 (deriving for each protein from the same gel), together with β-actin are shown. mRNA and protein levels were expressed as a percentage of the control group. Data are mean ± SEM (n = 7–8). Statistical analysis between groups was performed by one-way ANOVA, followed by LSD post-hoc analysis (*P* < 0.05), a ≠ b ≠ c.

The gene and protein expression levels of capsaicin receptor *Trpv1* and of *Ucp2* were also studied to ascertain potential mechanisms involved in the hepatic health benefits shown by capsaicin treatment alone, but not by the combination of hesperidin and capsaicin (Fig. 4). Animals treated with capsaicin showed greater *Trpv1* mRNA level than the controls and animals treated with hesperidin. However, animals treated with the combination displayed intermediate levels, similar to those of the WD group. No significant differences between groups were found concerning protein levels of TRPV1. Regarding *Ucp2*, animals treated with capsaicin displayed higher mRNA levels and higher UCP2 protein levels than the control, HESP and HESP + CAP groups (*P* < 0.05, LSD *post-hoc* analysis).

### Expression of energy metabolism-related genes in rWAT

**Figure 5:**
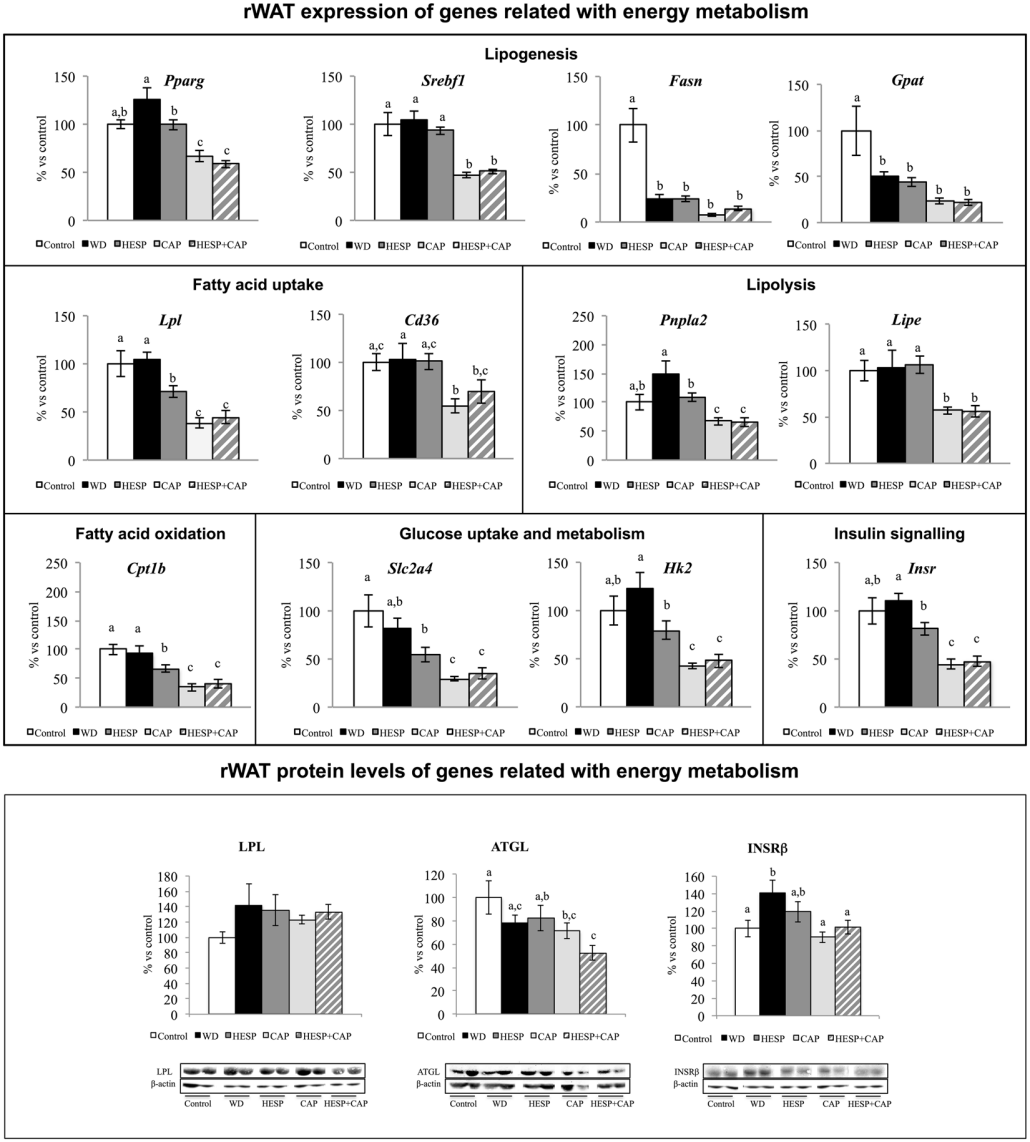
mRNA expression levels and protein levels of genes related to energy metabolism (lipogenesis, fatty acid uptake, lipolysis, fatty acid oxidation, glucose uptake and metabolism, and insulin signalling) in retroperitoneal white adipose tissue of control (C), western diet (WD), hesperidin (HESP), capsaicin (CAP), and hesperidin and capsaicin (HESP + CAP) rats at the end of treatment. mRNA levels were measured by real-time PCR. Specific protein levels were measured by western blot. Representative bands for LPL, ATGL and INSRβ (deriving for each protein from the same gel), together with β-actin are shown. mRNA and protein levels were expressed as a percentage of the control group. Data are mean ± SEM (n = 7–8). Statistical analysis between groups was performed by one-way ANOVA, followed by LSD post-hoc analysis (*P* < 0.05), a ≠ b ≠ c.

The mRNA levels of selected genes related to energy metabolism in the rWAT in the different groups of animals are showed in Fig. 5. The retroperitoneal depot was chosen as a representative of WAT because of its high metabolic activity compared to other depots^24^ and its relationship with the development of insulin resistance and type 2 diabetes^25^.

Animals treated with capsaicin or the combination of hesperidin and capsaicin displayed lower expression levels of the lipogenesis-related genes *Pparg* and *Srebf1* with respect to the WD group (*P* < 0.05, LSD *post-hoc* analysis). Animals treated with hesperidin also showed lower expression levels of *Pparg* with respect to the WD group, but the decrease was less marked than that occurring in the CAP and HESP + CAP groups (P < 0.05, LSD *post-hoc* analysis). No significant differences were found between the controls and the WD group concerning the expression levels of *Pparg* and *Srebf1*. *Fasn* and *Gpat* expression levels were decreased in all groups of animals under western diet feeding with respect to controls (*P* < 0.05, LSD *post-hoc* analysis), with no significance differences among them.

Regarding fatty acid uptake-related genes, HESP, CAP and HESP + CAP displayed lower expression levels of *Lpl* than the control and WD groups. The decrease in the CAP and HESP + CAP groups was more marked and significant than that occurring for the HESP group (*P* < 0.05, LSD *post-hoc* analysis). However, no significant differences between groups were found regarding LPL protein levels. The CAP and HESP + CAP groups also displayed a decrease in *Cd36* mRNA levels with respect to the WD group (*P* < 0.05, LSD *post-hoc* analysis) whereas no changes were observed in the HESP group. No significant differences were found between the controls and the WD group concerning the expression levels of both genes.

The CAP and HESP + CAP groups displayed lower expression levels of the lipolysis related genes *Pnpla2* and *Lipe* with respect to the control, WD and HESP groups (*P* < 0.05, LSD *post-hoc* analysis). The HESP group also showed lower mRNA levels of *Pnpla2* than the WD group, but the decrease was less marked than that occurring in the CAP and HESP + CAP groups (*P* < 0.05, LSD *post-hoc* analysis). No significant differences were found between the control and the WD groups concerning the expression levels of both genes. No significant differences between the control and the WD groups were either found regarding expression levels of ATGL protein, but animals of the HESP + CAP group displayed lower levels than the control and HESP groups (*P* < 0.05, LSD *post-hoc* analysis).

The expression of the fatty acid oxidation related gene *Cpt1b* showed a pattern of expression similar to that described for the *Lpl* in the CAP and HESP + CAP groups, and to a lesser extent the HESP group, showing lower expression levels than the control and WD groups (*P* < 0.05, LSD *post-hoc* analysis).

As far as glucose uptake and metabolism are concerned, the CAP and HESP + CAP groups also showed lower expression levels of the *Slc2a4* and *Hk2* genes when compared to the control, WD and HESP groups. In turn, the HESP group showed lower expression levels of *Slc2a4* than the controls and of *Hk2* than the WD group (*P* < 0.05, LSD *post-hoc* analysis). No significant differences were found between the control and the WD groups concerning the expression levels of both genes.

Regarding insulin signalling, the HESP, CAP and HESP + CAP groups showed reduced expression levels of the *Insr* gene with respect to the WD group (*P* < 0.05, LSD *post-hoc* analysis). The decrease was more marked in the CAP and HESP + CAP groups, which also showed lower expression levels than those of the HESP and the control groups. A similar pattern was observed regarding INSRβ protein levels. The CAP and HESP + CAP groups expressed lower INSRβ protein levels than animals of the WD group (*P* < 0.05, LSD *post-hoc* analysis), but no significant differences were found between the WD and CAP groups.

### *In vitro* assessment of the effects of hesperidin, capsaicin, and the combination of both compounds on *Trpv1* and *Ucp2* expression

**Figure 6:**
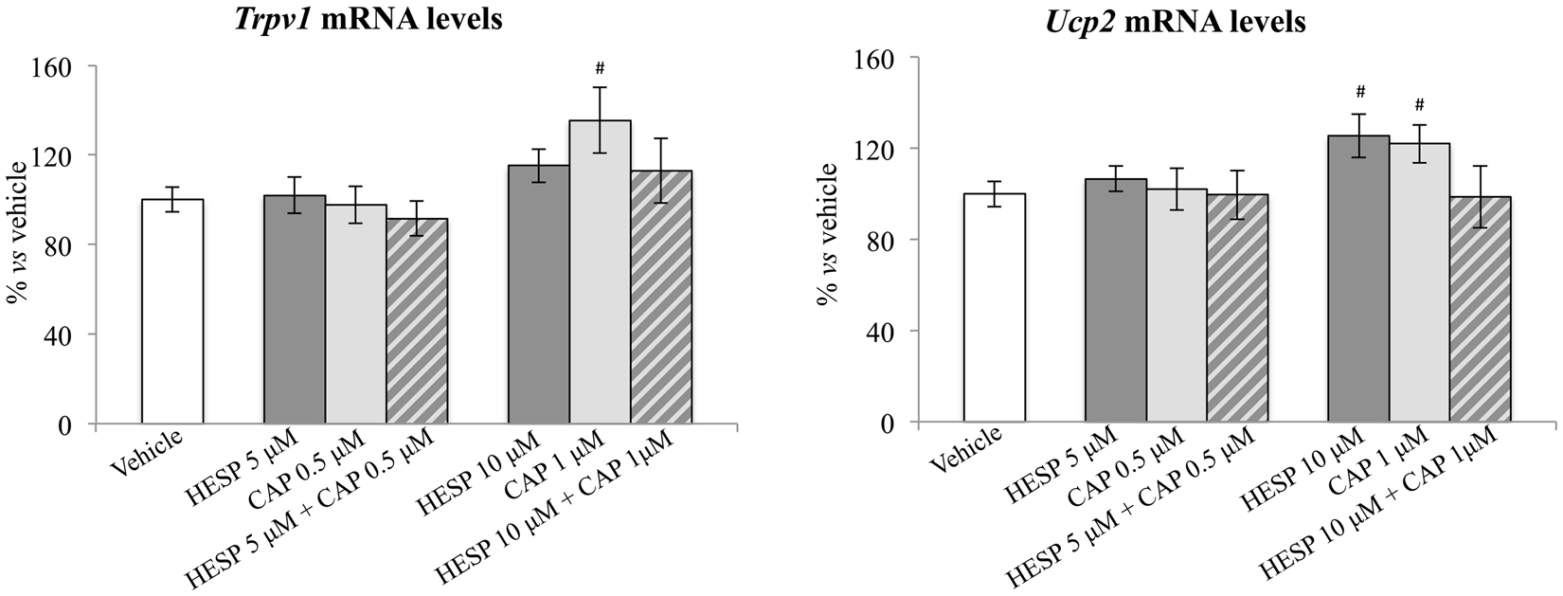
mRNA levels of *Trpv1* and *Ucp2* in HepG2 cells treated with hesperidin (HESP) (5 μmol/L and 10 μmol/L), capsaicin (CAP) (0.5 μmol/L and 1 μmol/L), the combination of both HESP + CAP, or the vehicle for 24 hours. mRNA levels were measured by real-time PCR and expressed as a percentage of the vehicle group. Data are mean ± SEM (n = 7–12). Non significant differences were found between groups by one-way ANOVA, followed by LSD post-hoc analysis (P < 0.05). ^#^Different from vehicle (*P* < 0.05, Student’s t test).

Direct effects of hesperidin and capsaicin, individually and in combination, on *mRNA* of *Trpv1* and *Ucp2* were assessed *in vitro* in the human hepatoma-derived cell line HepG2 (Fig. 6). Results showed that capsaicin (1 μmol/L) treatment, but not the combination of hesperidin (10 μmol/L) + capsaicin (1 μmol/L), resulted in higher *Trpv1* mRNA levels compared with the vehicle (*P* < 0.05, Student’s *t* test). No effects were found with the lower doses assayed (5 μmol/L hesperidin, 0.5 μmol/L capsaicin). Regarding *Ucp2*, both hesperidin (10 μmol/L) and capsaicin (1 μmol/L), separately, resulted in higher expression levels of *Ucp2* compared with the vehicle (*P* < 0.05, Student’s *t* test); however, no effect was found with the combination of both compounds. No significant effects were found with the lower doses studied of both compounds.

## Discussion

The prevalence of obesity and its related metabolic complications has increased in the last decades at an epidemic rate, becoming a serious global health problem^1^. Feeding behaviour, and particularly the intake of a high-fat, high-sucrose diet, the so called western diet, may be considered as one of the main determinants^2^. Besides nutritional advises, the development of food-related strategies to prevent or attenuate metabolic syndrome related consequences is of great interest.

Studies performed in last decades have pointed out the bioactive effects of the flavonoid hesperidin against obesity-related alterations, mainly because to its TG-lowering, anti-inflammatory, antioxidant, and insulin-sensitizing effects^5–9,26^. In turn, capsaicin has a demonstrated anti-obesity effect. It has been shown to reduce body weight gain, hepatic lipid accumulation and insulin resistance induced by high-fat diet feeding in animals^13,14^. Thus, both hesperidin and capsaicin might be promising bioactives for prevention or treatment of metabolic syndrome components, with potential complementary action. However, the potential benefits of the combination of both compounds preventing or ameliorating diet-induced alterations had not been explored yet. Here, we assessed the effects of hesperidin and capsaicin, alone and in combination, to prevent metabolic alterations related to the metabolic syndrome in rats exposed to a western diet.

As expected, the consumption of a western diet for 8 weeks in adult rats resulted in a higher body weight and body fat gain, and, thereby, the animals exhibited at the end of treatment greater fat content than animals fed standard diet. Moreover, western diet-fed animals displayed greater leptin levels and higher TG and NEFA levels in the fed state than the controls. Interestingly, capsaicin treatment (4 mg/kg/day) during 2 months in western diet-fed animals effectively reduced body fat gain, as well as fed-state levels of leptin and NEFA. In turn, hesperidin treatment (100 mg/kg/day) attenuated body fat increase, as well as the increase in circulating leptin levels associated to western diet feeding, but the effects were lower than those observed by capsaicin and did not reach statistical significance. Other authors have also found no significant effects of hesperidin on body weight in obese mice under low-fat diet, but significant effects were observed when hesperidin was combined with caffeine^27^. Notably, the combination of hesperidin and capsaicin did show lower effects on body fat gain than those induced by capsaicin alone; in fact, body fat percentage of these animals at the end of the treatment was similar to that of animals treated with hesperidin, and not different from that of western diet-fed animals.

The anti-obesity action of capsaicin (and non-pungent related compounds) has been previously described in both animal and human studies. Evidence suggests that capsaicin decreases body weight by increasing energy expenditure^28–32^, stimulating adipose tissue lipid mobilization and fat oxidation^30,31,33,34^, and reducing energy intake^29,32,35,36^; however, its concrete mechanism is not clear.

In the present study we show that animals receiving either hesperidin or capsaicin, separately, or the combination, along with a western diet, showed a trend to eat fewer calories than western diet-fed animals (decreases of 5.4%, 7.4% and 7.1%, respectively), reaching values not different from that of the controls. However, no additive effects were found with the combination of both compounds. Thus, the lower intake may contribute to the lower body fat gain of animals, more marked for the animals that received capsaicin. Nevertheless, in spite of the described effects of capsaicin increasing energy expenditure, we did not observe any significant enhancement in energy expenditure induced by capsaicin, or by hesperidin, at 6 weeks of treatment. The lack of significant effects might be tentatively explained by a reduced sympathetic responsiveness to capsaicin treatment in western diet fed-rats, as described in obese subjects^37^, which may impair diet-induced thermogenesis; however, the thermogenic effects of capsaicin in white and brown adipose tissues have not been directly explored here.

The effects of capsaicin in preventing fat accumulation may be explained, at least in part, by the effects on lipid metabolism in adipose tissue, particularly by its lipogenesis inhibitory effect^38^. In fact, animals treated with capsaicin displayed in rWAT decreased expression levels of lipogenesis-related genes (*Pparg* and *Srebf1*), along with decreased expression levels of genes related with fatty acid uptake (*Lpl*, *Cd36*) and oxidation (*Cpt1b*), lipolysis (*Pnpla2*, *Lipe*), and glucose uptake and metabolism (*Slc2a4*, *Hk2*). These effects may be tentatively related with a decreased insulin action in the adipose tissue, as deduced from the trend to lower insulin levels found in animals in the capsaicin group, together with the decreased mRNA levels of the *Insr* gene and of INSRβ protein levels with respect to animals in the WD group. Expression levels of some of the above-mentioned genes (*Pparg*, *Lpl*, *Pnpla2*, *Cpt1b*, *Hk2*, and *Insr*) were also diminished in animals treated with hesperidin, with respect to western diet-fed animals, but the effects were generally less marked than those observed with capsaicin, in accordance with the more modest effect of hesperidin on body fat gain. The combination of capsaicin and hesperidin also elicited no additional effects to those observed with capsaicin alone, with the exception of ATGL, whose protein levels were only significantly lower in the group treated with the combination of compounds with respect to the control group.

According to the improvement of lipid metabolism in adipose tissue, capsaicin treatment also showed interesting effects in preventing diet-induced insulin resistance. Capsaicin treatment prevented the increase in HOMA-IR in western diet-fed rats, but notably, the effects were reduced when capsaicin was combined with hesperidin. The capacity of capsaicin in ameliorating insulin resistance has been previously described in rodents^13^. Several studies in humans have also revealed that capsaicin has a modest effect in type 2 diabetes^39^. Unlike capsaicin, no significant effects were observed for hesperidin on insulin resistance in western diet-fed rats.

Treatment with either hesperidin or capsaicin alone, but not with the combination of both bioactives, attenuated the increase of circulating TG levels in the fed-state occurring in western diet-fed rats. In addition, both compounds separately, and to a lesser extent the combination, attenuated hepatic lipid increase due to western diet feeding. Moreover, histological analysis unveiled that both hesperidin and capsaicin, when administered alone, conferred protective effect on the development of non-alcoholic fatty liver disease (NAFLD) and prevented the features of non-alcoholic steatohepatitis (NASH), such as hepatocyte ballooning, necrotic hepatocytes and infiltrated lymphocytes, found in western diet-fed animals. However, some signs of NASH were found in animals treated with the combination of both bioactives. Despite this, no significant differences were found between groups in the plasma levels of the liver damage markers AST and ALT. A sex-dependent effect has been described regarding the risk of developing NAFLD and NASH in animal and human studies, since males are at an increased risk compared with females^40^. Therefore, one of the limitations of the present study is that this has only been performed in male rats. This must be considered when extrapolating the results to humans.

Hesperidin has been previously described to reduce serum TG in animal models^41–43^ and hypertriglyceridemic subjects^7^. The mechanisms involved in the TG-lowering effects of hesperidin have been reported to be the reduction of hepatic TG content through inhibition of lipogenesis and induction of fatty acid oxidation^43^, and the downregulation of synthesis and secretion of very-low-density lipoproteins (VLDL)^44^. According to this notion, we observed here that hesperidin-treated animals displayed decreased expression levels of three key lipogenesis-related genes, *Srebf1*, *Fasn* and *Scd1*, as well as normalisation of *Pklr* gene expression to control levels. In addition, these animals exhibited decreased hepatic expression levels of the *Insr* and *Lepr* genes, compared to levels of expression in western diet-fed rats, suggesting a decreased lipogenic action of insulin, along with decreased fatty oxidation activity. Although there is no clear consensus in the literature on the effects of leptin in liver^45^, it has been proposed that leptin may have insulin sensitizing effects, controlling the extent of insulin action on this tissue^46^. Notably, disruption of hepatic leptin signalling has been shown to protect mice from diet-related glucose intolerance^46^. Therefore, it is suggested that the decreased hepatic lipogenesis in hesperidin-treated animals, associated to a decreased insulin action on this tissue, may be one of the mechanisms whereby this bioactive protects against diet-induced hepatic pathologies.

In turn, capsaicin treatment elicited little effects on the expression of hepatic lipogenesis-related genes compared to changes observed with hesperidin. It has been reported that capsaicin regulates hepatic lipid metabolism and prevent lipid deposition in liver through TRPV1 activation^19^. Capsaicin action on TRPV1 involves up-regulation of UCP2, a mitochondrial membrane transporter that can provide fatty acid translocation, preventing its accumulation in the mitochondrial matrix^19^. Here, the presence of higher mRNA levels of *Ucp2* and of the encoded protein UCP2 in capsaicin-treated animals compared to the other groups is in accordance with the involvement of UCP2 in the protective effects of capsaicin on lipid deposition in liver, providing protective effects from hepatocellular lipotoxicity^47^. Therefore, both capsaicin and hesperidin, separately, are able to overcome the effects of western diet on hepatic lipid metabolism, and hence ameliorate hepatic steatosis and prevent NASH associated with western diet consumption. Unexpectedly, the effects were blunted or even negligible when animals were treated with both compounds simultaneously. Mechanisms underlying this interaction are unknown, but the comparison of gene expression patterns in liver has given some clues. On the one hand, capsaicin treatment blocked the decrease in the hepatic expression of *Lepr* induced by hesperidin, which may be tentatively associated with an increase in insulin-induced lipogenesis activity in this tissue. In fact, the combination of both compounds up-regulated the expression of the lipogenic gene *Gadph*, reaching levels higher than those of animals treated with hesperidin alone. On the other hand, hesperidin seems to override the effects of capsaicin through its TRPV1 receptor. The induction of *Trpv1* expression by capsaicin was attenuated with the simultaneous treatment with hesperidin, suggesting that the responsiveness to the capsaicin action was blunted in animals treated with the combination of bioactives. Accordingly, animals treated with the combination of bioactives did not show the increase in *Ucp2* mRNA levels or in UCP2 protein levels characteristic of capsaicin treated animals. Thus, it is suggested that both bioactives may mutually impair their ways of action on the improvement of liver health in western diet-fed animals. This hypothesis is supported by the results of an *in vitro* assay in the human liver HepG2 cell line, showing that the combination of hesperidin and capsaicin, blunts the effects of capsaicin on the increase of *Trpv1* and *Ucp2* expression levels.

Finally, regarding other components of the metabolic syndrome, results of the present study also bring evidence supporting the hypotensive effects of hesperidin and, to a lesser extent, of capsaicin. In fact, systolic and diastolic blood pressure was decreased in hesperidin treated animals, compared to untreated western diet-fed rats, reaching levels lower than the controls. Animals treated with capsaicin or the combination of hesperidin and capsaicin also exhibited lower diastolic blood pressure than western diet-fed animals, and similar to control animals. This suggests interesting anti-hypertension effects of hesperidin, beyond their potential fat-lowering effects. The hypotensive effect of hesperidin has been previously described both at short- and long-term treatments in models of hypertensive rats^48,49^, and this has been related to the capacity to improve endothelium-dependent vasorelaxation by increasing the availability of nitric oxide^50,51^.

In summary, the results of the present study show that capsaicin and hesperidin, separately, exhibit different effects on fat accumulation and metabolic syndrome-related disorders in rats fed on western diet. More precisely, capsaicin induces protective phenotype against obesity, decreasing body fat gain and preventing insulin resistance under western diet feeding, whereas hesperidin has little effects on body fat gain and no apparent effects on insulin resistance. No additive effects, or even blunted with respect to those observed with capsaicin, were observed with the combination of both bioactives. However, capsaicin and hesperidin alone, improve blood lipid profile, diminish hepatic lipid accumulation, and prevent NASH in western diet-fed rats, although the effects are mitigated or even annulled with the combination of both compounds. In turn, hesperidin alone, and to a lesser extent capsaicin or the combination of hesperidin plus capsaicin, display hypotensive effects in western diet-fed rats. Therefore, these results give additional evidence supporting that intervention with either capsaicin or hesperidin alone may be promising in populations at high risk of metabolic syndrome-related alterations, particularly fatty liver disease. However, negative results found with the combination of both compounds deserve to be taken into account when considering potential mixtures of bioactives as strategies for obesity prevention.

## Methods

### Animals and experimental design

The animal protocol followed was reviewed and approved by the Bioethical Committee of the University of the Balearic Islands (Resolution Number 7619, October, 2015) and guidelines for the use and care of laboratory animals of the University were followed.

The study was performed in 39 three-month-old male Wistar rats randomly divided into 5 groups: Control (n = 7), animals fed with a standard chow diet (3.3 kcal/g, with 19% kcal from protein, 73% from carbohydrate, and 8% from fat) (Pan-lab, Barcelona, Spain); WD (n = 8), animals fed with a high-fat, high-sucrose diet (western diet, 4.7 kcal/g, with 17% kcal from protein, 43% from carbohydrate, and 41% from fat) (Research Diets, Inn, New Brunswick, NJ, USA); HESP (n = 8), animals fed with a western diet and treated with hesperidin (100 mg/kg/day); CAP (n = 8), animals fed a western diet and treated with capsaicin (4 mg/kg/day); and HESP + CAP (n = 8), animals fed with a western diet and treated with the combination of hesperidin (100 mg/kg/day) and capsaicin (4 mg/kg/day). Hesperidin and capsaicin were purchased from Aldrich Co. LLC. (St. Louis, MO). They were prepared in 0.9% saline and administered orally, by gavage, once a day after the beginning of the light cycle (8:00 h). Control and WD groups received the same volume of saline (1 mL/kg). The animals were kept with this treatment for 8 weeks, until they were 5 months old. All rats were individually housed under controlled temperature (22 °C) and a 12 hours light-dark cycle, and had unlimited access to tap water and standard diet or western diet, depending on the group.

Body weight and body composition (by EchoMRI-700TM, Echo Medical Systems, LLC., TX, USA) were measured at baseline and at the end of the treatment (week 8). Blood samples were obtained on week 7 after 12 hours fasting from saphenous vein and at sacrifice under feeding conditions (truncal blood). Blood samples were collected in heparinized containers, then centrifuged at 1000 *g* for 10 min to obtain the plasma, and stored at 20 °C until analysis. After 8 weeks of treatment, animals were sacrificed by decapitation under fed condition. Retroperitoneal white adipose tissue (rWAT) and the liver were rapidly removed, weighted, frozen in liquid nitrogen, and stored at −80 °C until subsequent studies.

### Indirect calorimetry and locomotive activity measurements

Animals were monitored for 24 hours to assess energy expenditure by indirect calorimetry and locomotive activity by using the LabMaster-CalSys-Calorimetry System (TSE Systems, Bad Homburg, Germany) after 6 weeks of treatment. In order to reduce potential stress, animals were individually housed and acclimated to the respiratory cages for 24 hours before the measurement began. Data on gas exchanges (VO_2_; ml kg ^−1^ h^−1^ and VCO_2_; ml kg ^−1^ h^−1^) were measured via an open circuit indirect calorimetry system for 24 hours Rates of oxygen consumption and carbon dioxide production were monitored for 5 min every 45 min for each animal or reference cage (our system can handle 8 animal cages and 1 reference cage, simultaneously). Mean energy expenditure (kcal/h) and respiratory exchange ratio (RER) values were calculated over 24 hours. Locomotive activity (counts/h) was measured continuously by an infrared beam system integrated in the LabMaster System for 24 hours.

### Blood pressure measurement

Systolic and diastolic blood pressure was measured after 5 weeks of treatment. This was determined without anaesthesia using non-invasive blood pressure methodology. It consists of using a tail-cuff sphygmomanometer with a photoelectric sensor (Niprem 546, Cibertec S.A., Spain) placed on the animal’s tail to occlude the blood flow. Niprem software V1.8 was used. All measurements were carried out between 1600 and 1900 h. For each animal, systolic and diastolic blood pressure values were calculated as the mean of five measurements.

### Measurement of circulating parameters

Fresh blood glucose concentration was measured with an Accu-Chek Glucometer (Roche Diagnostics, Barcelona, Spain). Commercial rat ELISA kits were used for the quantification of circulating plasma levels of insulin (inter- and intra-assay coefficient of variation were in the range between 2.8–5.1% and 3.2–10%, respectively, Mercodia AB, Uppsala, Sweden), and leptin (inter- and intra-assay coefficient of variation were in the range between 3.3–4.3% and 4.7–7.6%, respectively, R&D Systems, Minneapolis, MN, USA). Commercial enzymatic kits were used for determination of plasma levels of TG (Triglyceride (INT) 20, (Sigma-Aldrich Co., LLC, Madrid, Spain), non-esterified fatty acids (NEFA) (Wako Chemicals GmbH, Neuss, Germany), and aspartate transaminase (AST) and alanine transaminase (ALT) (QCA, Química Clínica Aplicada S.A., Tarragona, Spain), each one according to the manufacturer’s instructions. The homeostatic model assessment for insulin resistance (HOMA-IR) was used to assess insulin resistance according to the formula described by Matthews and collaborators^52^.

### Quantification of hepatic lipid content

Total lipids were extracted from about 600 mg of hepatic tissue and quantified by the method of Folch *et al*.^53^.

### Histological analysis

Liver samples of 5 animals per group were used for histological analysis. Liver tissue samples were fixed by immersion in 4% paraformaldehyde in 0.1 M sodium phosphate buffer (pH 7.4) overnight at 4 °C. Following, they were washed in phosphate buffer, dehydrated in a graded series of ethanol, cleared in xylene and embedded in paraffin blocks. Five-micrometer-thick sections of tissues were cut with a microtome and mounted on slides.

Liver sections were classified into four grades depending on fat accumulation following Burnt *et al*. classification^21^: grade 0 was assigned when there was no fat accumulation; grade 1 when fat vacuoles were observed in less than 33% of hepatocytes; grade 2 when 33–66% of hepatocytes contained fat vacuoles; and grade 3 when they were found in more than 66% of hepatocytes.

### RNA Extraction

Total RNA was extracted from the liver by Tripure Reagent (Roche Diagnostic Gmbh, Mannheim, Germany) and from retroperitoneal white adipose tissue (rWAT) by an E.Z.N.A. RNA purification system (Omega Biotek, Inc., Norcross, GA) each one according to the manufacturer’s instructions. Isolated RNA was quantified using NanoDrop ND-1000 spectrophotometer (NanoDrop Technologies Wilmington, DE, USA). Its integrity was confirmed using agarose gel electrophoresis.

### Real-time quantitative PCR analysis

Real-time polymerase chain reaction was used to measure mRNA expression levels of selected genes in the liver and rWAT. Precisely, in liver: sterol regulatory element binding transcription factor 1 (*Srebf1*), fatty acid synthase (*Fasn*), sterol coenzyme A desaturase (*Scd1*), glyceraldehyde 3-phosphate dehydrogenase (*Gadph)*, carnitine palmitoyltransferase 1a, liver (*Cpt1a*), peroxisome proliferator activated receptor alpha (*Ppara*), fibroblast growth factor 21 (*Fgf21*), Glucokinase (*Gck*), pyruvate kinase (*Pklr*), insulin receptor (*Insr*), insulin receptor substrate 1 (*Irs1*), leptin receptor (*Lepr*), transient receptor potential cation channel, subfamily V, member 1 (*Trpv1*), and uncoupling protein 2 (*Ucp2*). In WAT: peroxisome proliferator activated receptor gamma (*Pparg*), *Srebf1, Fasn*, glycerol-3-phosphate acyltransferase (*Gpat*), lipoprotein lipase (*Lpl*), CD36 molecule (*Cd36*), patatin-like phospholipase domain containing 2 (*Pnpla2*), hormone-sensitive lipase (*Lipe*), carnitine palmitoyltransferase 1b, muscle isoform (*Cpt1b*), glucose transporter 4 (*Slc2a4*), hexokinase II (*HK2*), and *Insr*. Guanosine Diphosphate Dissociation Inhibitor (*Gdi*) was used as a reference gene. All primers were obtained from Sigma Genosys (Sigma Aldrich Co., LLC, Madrid, Spain).

Total RNA (0.25 μg, in a final volume of 5 μL) was denatured at 65 °C for 10 min and then reverse transcribed to cDNA using MuLV reverse transcriptase (Applied Biosystems, Madrid, Spain) at 20 °C for 15 min and 42 °C for 30 min with a final step of 5 min at 95 °C in an Applied Biosystems 2720 Thermal Cycler (Applied Biosystems). Each polymerase chain reaction (PCR) was performed from diluted (1/20) cDNA template, forward and reverse primers (1 μmol/L each), and Power SYBER Green PCR Master Mix (Applied Biosystems, Foster City, CA). Real-time PCR was performed using the Applied Biosystems StepOnePlus real-time PCR system with the following profile: 10 min at 95 °C, followed by a total of 40 two-temperature cycles (15 s at 95 °C and 1 min at 60 °C). To verify the purity of the products, a melting curve was produced after each run according to the manufacturer’s instructions. The threshold cycle (Ct) was calculated by the instrument’s software (StepOne Software v2.2.2), and the relative expression of each mRNA was calculated as a percentage of male control rats using de 2^−ΔΔCt^ method.

### Western blot analysis

Protein levels of adipose triglyceride lipase (ATGL) (Cayman, Michigan, USA), CPT1 (Santa Cruz Biotechnology, Inc., Heidelberg, Germany), LPL (Santa Cruz Biotechnology, Inc.), INSRβ (Cell Signaling Technology, Leiden, The Netherlands), TRPV1 (Thermo Fisher Scientific, Madrid, Spain) and UCP2 (Abcam, Cambridge, UK) in liver or rWAT were determined by western blot. Tissues were homogenized at 4 °C in 1:10 (w:v) for liver and 1:2 (w:v) for rWAT of phosphate buffer saline, and then processed for western blot as previously described^54^ with some modifications. For analysis, 50 or 60 µg total protein was fractionated by a 10% or a 4–15% SDS-PAGE (Criterion^TM^TGX^TM^, Bio-Rad Laboratories, Madrid, Spain) and then electrotransferred onto nitrocellulose membrane (Bio-Rad Laboratories). After blocking, membranes were incubated with the corresponding primary antibody, and then with the infrared-dyed secondary anti-IgG antibody (LI-COR Biociences, Nebraska, USA). For IR detection, membranes were scanned in Odyssey Infrared Imaging System (LI-COR Biociences) and bands were quantified using Odyssey Software V.3.0 (LI-COR Biosciences). Housekeeping protein used was β-Actin (Cell Signaling Technology, Leiden, The Netherlands).

### Cell culture and treatments

HepG2 cells, a human hepatoma-derived cell line, were obtained from ATCC (American Type Culture Collection; LGC Deselaers SL, Barcelona, Spain). They were grown in eagles minimum essential medium (EMEM) (ATCC) supplemented with 10% (v/v) fetal bovine serum (Gibco, Thermofisher Scientific, Madrid, Spain) and antibiotics (50 IU penicillin/mL and 50 mg streptomycin/mL) at 37 °C, in a humidified atmosphere with 5% CO_2_. The cells (approximately 3 × 10^3^ cells per cm^2^) were plated in 24-well cell culture plates and cultured for 10 days, with medium changes every 3–4 days. When the culture cells reached approximately 90% confluence, they were treated for 24 h with 0.5 and 1 μmol/L of capsaicin (Sigma Aldrich, St. Louis, MO, USA), 5 and 10 μmol/L of hesperidin (Sigma Aldrich, St. Louis, MO, USA), and the mixture of both compounds (0.5 μmol/L capsaicin + 5 μmol/L hesperidin, and 1 μmol/L capsaicin + 10 μmol/L hesperidin). The concentrations of capsaicin assayed were selected from the literature. 1 μmol/L capsaicin has been shown to stimulate *Trpv1* expression in porcine iliac artery endothelial cells^55^. Regarding hesperidin, the concentrations were selected to keep the proportion with respect to capsaicin tested in the *in vivo* study. Capsaicin was prepared in ethanol and hesperidin in water. All wells received the same amount of water and ethanol. After treatment, total RNA was extracted with E.Z.N.A. RNA purification system, and mRNA levels of *Trpv1* and *Ucp2* were measured as previously described^56^. 

### Statistical Analysis

All data are expressed as the mean ± Standard Error of the Mean (SEM). Data were checked for normality using Shapiro-Wilks normality test. For multi-group comparisons Levene’s test was performed to assess whether the variance is equal between groups; if the variance was heterogeneous, data were log-transformed before analysis. Differences among groups were assessed by one-way ANOVA followed by least significances difference (LSD) *post-hoc* comparison. The effect of fasting versus *ad libitum* feeding in blood parameters was assessed with a paired *t* test. Student’s *t* test was performed to assess differences in gene expression in *in vitro* studies to compare the different treatments *versus* the vehicle. The analyses were performed with IBM SPSSP Statistics 21. Threshold of significance was defined at *P* < 0.05.

## References

[1] WHO. Obesity and overweight. Fact sheet N°311. Updated oct 2017, http://www.who.int/mediacentre/factsheets/f311/en/.

[2] Cordain L (2005). Origins and evolution of the Western diet: health implications for the 21st century. Am J Clin Nutr.

[3] Chan RS, Woo J (2010). Prevention of overweight and obesity: how effective is the current public health approach. Int J Environ Res Public Health.

[4] Redan BW, Buhman KK, Novotny JA, Ferruzzi MG (2016). Altered Transport and Metabolism of Phenolic Compounds in Obesity and Diabetes: Implications for Functional Food Development and Assessment. Adv Nutr.

[5] Peng H (2016). Inhibition of Fat Accumulation by Hesperidin in Caenorhabditis elegans. J Agric Food Chem.

[6] Assini JM, Mulvihill EE, Huff MW (2013). Citrus flavonoids and lipid metabolism. Curr Opin Lipidol.

[7] Miwa Y (2005). Glucosyl hesperidin lowers serum triglyceride level in hypertriglyceridemic subjects through the improvement of very low-density lipoprotein metabolic abnormality. J Nutr Sci Vitaminol (Tokyo).

[8] Bok SH (1999). Plasma and hepatic cholesterol and hepatic activities of 3-hydroxy-3-methyl-glutaryl-CoA reductase and acyl CoA: cholesterol transferase are lower in rats fed citrus peel extract or a mixture of citrus bioflavonoids. J Nutr.

[9] Li C, Schluesener H (2017). Health-promoting effects of the citrus flavanone hesperidin. Crit Rev Food Sci Nutr.

[10] Whiting S, Derbyshire E, Tiwari BK (2012). Capsaicinoids and capsinoids. A potential role for weight management? A systematic review of the evidence. Appetite.

[11] Thiele R, Mueller-Seitz E, Petz M (2008). Chili pepper fruits: presumed precursors of fatty acids characteristic for capsaicinoids. J Agric Food Chem.

[12] Sharma SK, Vij AS, Sharma M (2013). Mechanisms and clinical uses of capsaicin. Eur J Pharmacol.

[13] Kang JH (2010). Dietary capsaicin reduces obesity-induced insulin resistance and hepatic steatosis in obese mice fed a high-fat diet. Obesity (Silver Spring).

[14] Leung FW (2014). Capsaicin as an anti-obesity drug. Prog Drug Res.

[15] Zheng Jia, Zheng Sheng, Feng Qianyun, Zhang Qian, Xiao Xinhua (2017). Dietary capsaicin and its anti-obesity potency: from mechanism to clinical implications. Bioscience Reports.

[16] Zhang LL (2007). Activation of transient receptor potential vanilloid type-1 channel prevents adipogenesis and obesity. Circ Res.

[17] Montell C, Birnbaumer L, Flockerzi V (2002). The TRP channels, a remarkably functional family. Cell.

[18] Fernandes ES, Fernandes MA, Keeble JE (2012). The functions of TRPA1 and TRPV1: moving away from sensory nerves. Br J Pharmacol.

[19] Li L (2012). TRPV1 activation prevents nonalcoholic fatty liver through UCP2 upregulation in mice. Pflugers Arch.

[20] Akabori H (2007). Transient receptor potential vanilloid 1 antagonist, capsazepine, improves survival in a rat hemorrhagic shock model. Ann Surg.

[21] Rodríguez A (2012). Short-term effects of sleeve gastrectomy and caloric restriction on blood pressure in diet-induced obese rats. Obes Surg.

[22] Schreuder MF, Fodor M, van Wijk JA, Delemarre-van de Waal HA (2007). Weekend versus working day: differences in telemetric blood pressure in male Wistar rats. Lab Anim.

[23] Brunt EM, Janney CG, Di Bisceglie AM, Neuschwander-Tetri BA, Bacon BR (1999). Nonalcoholic steatohepatitis: a proposal for grading and staging the histological lesions. Am J Gastroenterol.

[24] Palou M (2010). Regional differences in the expression of genes involved in lipid metabolism in adipose tissue in response to short- and medium-term fasting and refeeding. J Nutr Biochem.

[25] Gabriely I (2002). Removal of visceral fat prevents insulin resistance and glucose intolerance of aging: an adipokine-mediated process?. Diabetes.

[26] Mosqueda-Solís A (2017). Screening of potential anti-adipogenic effects of phenolic compounds showing different chemical structure in 3T3-L1 preadipocytes. Food Funct.

[27] Ohara T, Muroyama K, Yamamoto Y, Murosaki S (2015). A combination of glucosyl hesperidin and caffeine exhibits an anti-obesity effect by inhibition of hepatic lipogenesis in mice. Phytother Res.

[28] Kawada T, Watanabe T, Takaishi T, Tanaka T, Iwai K (1986). Capsaicin-induced beta-adrenergic action on energy metabolism in rats: influence of capsaicin on oxygen consumption, the respiratory quotient, and substrate utilization. Proc Soc Exp Biol Med.

[29] Watanabe T, Kawada T, Yamamoto M, Iwai K (1987). Capsaicin, a pungent principle of hot red pepper, evokes catecholamine secretion from the adrenal medulla of anesthetized rats. Biochem Biophys Res Commun.

[30] Josse AR (2010). Effects of capsinoid ingestion on energy expenditure and lipid oxidation at rest and during exercise. Nutr Metab (Lond).

[31] Lee TA, Li Z, Zerlin A, Heber D (2010). Effects of dihydrocapsiate on adaptive and diet-induced thermogenesis with a high protein very low calorie diet: a randomized control trial. Nutr Metab (Lond).

[32] Ludy MJ, Mattes RD (2011). The effects of hedonically acceptable red pepper doses on thermogenesis and appetite. Physiol Behav.

[33] Kawada T, Hagihara K, Iwai K (1986). Effects of capsaicin on lipid metabolism in rats fed a high fat diet. J Nutr.

[34] Lejeune MP, Kovacs EM, Westerterp-Plantenga MS (2003). Effect of capsaicin on substrate oxidation and weight maintenance after modest body-weight loss in human subjects. Br J Nutr.

[35] Westerterp-Plantenga M S, Smeets A, Lejeune M P G (2004). Sensory and gastrointestinal satiety effects of capsaicin on food intake. International Journal of Obesity.

[36] Yoshioka M (1999). Effects of red pepper on appetite and energy intake. Br J Nutr.

[37] Matsumoto T (2000). Effects of capsaicin-containing yellow curry sauce on sympathetic nervous system activity and diet-induced thermogenesis in lean and obese young women. J Nutr Sci Vitaminol (Tokyo).

[38] Hong Q, Xia C, Xiangying H, Quan Y (2015). Capsinoids suppress fat accumulation via lipid metabolism. Mol Med Rep.

[39] Effect of treatment with capsaicin on daily activities of patients with painful diabetic neuropathy. Capsaicin Study Group. *Diabetes Care* **15**, 159–165 (1992). 10.2337/diacare.15.2.159 1547671

[40] Ballestri S (2017). NAFLD as a Sexual Dimorphic Disease: Role of Gender and Reproductive Status in the Development and Progression of Nonalcoholic Fatty Liver Disease and Inherent Cardiovascular Risk. Adv Ther.

[41] Akiyama S (2009). Hypoglycemic and hypolipidemic effects of hesperidin and cyclodextrin-clathrated hesperetin in Goto-Kakizaki rats with type 2 diabetes. Biosci Biotechnol Biochem.

[42] Chiba H (2003). Hesperidin, a citrus flavonoid, inhibits bone loss and decreases serum and hepatic lipids in ovariectomized mice. J Nutr.

[43] HY-YA, M., Arai, N., Sadakiyo, T. & Kubota, M. Glucosyl hesperidin lowers serum triglyceride level in the rats fed a high-fat diet through the reduction of hepatic triglyceride and cholesteryl ester. *Jpn Pharmacol Ther* **39**, 727–740 (2011).

[44] Miwa Y (2006). Suppression of apolipoprotein B secretion from HepG2 cells by glucosyl hesperidin. J Nutr Sci Vitaminol (Tokyo).

[45] Ceddia RB, Koistinen HA, Zierath JR, Sweeney G (2002). Analysis of paradoxical observations on the association between leptin and insulin resistance. FASEB J.

[46] Huynh FK (2010). Disruption of hepatic leptin signaling protects mice from age- and diet-related glucose intolerance. Diabetes.

[47] Baffy G (2005). Uncoupling protein-2 and non-alcoholic fatty liver disease. Front Biosci.

[48] Yamamoto M, Suzuki A, Hase T (2008). Short-term effects of glucosyl hesperidin and hesperetin on blood pressure and vascular endothelial function in spontaneously hypertensive rats. J Nutr Sci Vitaminol (Tokyo).

[49] Ohtsuki K (2002). Effects of long-term administration of hesperidin and glucosyl hesperidin to spontaneously hypertensive rats. J Nutr Sci Vitaminol (Tokyo).

[50] Rizza S (2011). Citrus polyphenol hesperidin stimulates production of nitric oxide in endothelial cells while improving endothelial function and reducing inflammatory markers in patients with metabolic syndrome. J Clin Endocrinol Metab.

[51] Yamamoto M (2013). Hesperidin metabolite hesperetin-7-O-glucuronide, but not hesperetin-3′-O-glucuronide, exerts hypotensive, vasodilatory, and anti-inflammatory activities. Food Funct.

[52] Matthews DR (1985). Homeostasis model assessment: insulin resistance and beta-cell function from fasting plasma glucose and insulin concentrations in man. Diabetologia.

[53] Folch J, Lees M, Sloane Stanley GH (1957). A simple method for the isolation and purification of total lipides from animal tissues. J Biol Chem.

[54] Palou M (2015). Moderate calorie restriction during gestation programs offspring for lower BAT thermogenic capacity driven by thyroid and sympathetic signaling. Int J Obes (Lond).

[55] Sun J (2013). TRPV1-mediated UCP2 upregulation ameliorates hyperglycemia-induced endothelial dysfunction. Cardiovasc Diabetol.

[56] Konieczna, J., Sánchez, J., Palou, M., Picó, C. & Palou, A. Blood cell transcriptomic-based early biomarkers of adverse programming effects of gestational calorie restriction and their reversibility by leptin supplementation. *Scientific Reports* **5**(1) (2015). 10.1038/srep09088 PMC4357898 25766068

