# Author Correction: Hesperidin and capsaicin, but not the combination, prevent hepatic steatosis and other metabolic syndrome-related alterations in western diet-fed rats

**DOI:** 10.1038/s41598-020-60110-6

**Published:** 2020-02-18

**Authors:** Andrea Mosqueda-Solís, Juana Sánchez, Bárbara Reynés, Mariona Palou, María P. Portillo, Andreu Palou, Catalina Picó

**Affiliations:** 1 Laboratory of Molecular Biology, Nutrition and Biotechnology (Nutrigenomics and Obesity group), University of the Balearic Islands, 07122 Palma, Spain; 2 Instituto de Investigación Sanitaria Illes Balears, 07010 Palma, Spain; 3 Nutrition and Obesity Group, Department of Nutrition and Food Science, Faculty of Pharmacy and Lucio Lascaray Research Center, University of the Basque Country (UPV/EHU), Vitoria, Spain; 4 CIBER Fisiopatología de la Obesidad y Nutrición (CIBEROBN), Instituto de Salud Carlos III (ISCIII), Madrid, Spain

Correction to: *Scientific Reports* 10.1038/s41598-018-32875-4, published online 10 October 2018

The Acknowledgements section in this Article is incomplete.

“This work was supported by the Spanish Government (AGL2015-67019-P), and the Instituto de Salud Carlos III, Centro de Investigación Biomédica en Red Fisiopatología de la Obesidad y Nutrición, CIBERobn. Laboratory of Molecular Biology, Nutrition and Biotechnology is a member of the European Research Network of Excellence NuGO (The European Nutrigenomics Organization, EU Contract: no. FP6-506360). A. Mosqueda-Solís is a recipient of a doctoral fellowship from the CONACYT (Mexico).”

should read:

“This work was supported by the Spanish Government (AGL2015-67019-P; MINECO/FEDER, UE), and the Instituto de Salud Carlos III, Centro de Investigación Biomédica en Red Fisiopatología de la Obesidad y Nutrición, CIBERobn. Laboratory of Molecular Biology, Nutrition and Biotechnology is a member of the European Research Network of Excellence NuGO (The European Nutrigenomics Organization, EU Contract: no. FP6-506360). A. Mosqueda-Solís is a recipient of a doctoral fellowship from the CONACYT (Mexico).”

